# Weekly measurement of HMGB1 during neoadjuvant chemoradiotherapy in rectal cancer patients: correlations with MRI response and ctDNA

**DOI:** 10.2478/raon-2026-0033

**Published:** 2026-06-26

**Authors:** Franziska Eckert, Vlatko Potkrajcic, Stephan Clasen, Elgin Hoffmann, Julian Taugner, Olga Seibel-Kelemen, Christopher Schroeder, Maximilian Niyazi, Kerstin Clasen

**Affiliations:** 1 Department of Radiation Oncology, University Hospital Tübingen, Tübingen, Germany; 2 Department of Radiation Oncology, Medical University Vienna, CCC Vienna, Vienna, Austria; 3 Department of Diagnostic and Interventional Radiology, District Hospital Reutlingen, Reutlingen, Germany; 4 Institute of Medical Genetics and Applied Genomics, University Hospital Tübingen, Tübingen, Germany; 5 RadioChirurgicum, CyberKnife Südwest, Göppingen, Germany

**Keywords:** High Mobility Group Box 1 (HMGB1) protein, radiotherapy, immunogenic cell death, magnetic resonance imaging, organ preservation, cell-free tumor DNA

## Abstract

**Background:**

Biomarkers are desirable to characterize individual tumors and to personalize multimodal treatment in locally advanced rectal cancer patients. In this regard, monitoring of High Mobility Group Box 1 (HMGB1) protein in blood samples is of interest in radiotherapy as HMGB1 is a consensus marker for immunogenic cell death (ICD) during anticancer treatment.

**Patients and methods:**

Plasma levels of HMGB1 were monitored weekly during long-term neoadjuvant chemoradiotherapy in 19 patients with locally advanced rectal cancer. HMGB1 levels were correlated with outcome and tumor volumes in magnetic resonance imaging (MRI) pre-therapeutically as well as in week 2 and week 5 of treatment. Furthermore, the association with circulating cell-free tumor DNA (ctDNA) was evaluated.

**Results:**

Higher pre-therapeutic levels of HMGB1 correlated positively with bigger initial MRI volumes and higher allele frequencies of ctDNA in the corresponding baseline blood sample. Courses of HMGB1 during treatment were variable and mostly undulating. Predominant ascent was observed in four patients who showed poor pathologic response. Declining levels in week 2 / 3 were associated with bigger percentage decrease of MRI tumor volume at the end of week 2.

**Conclusions:**

HMGB1 seems to be associated mostly with tumor burden as indicated by correlation with MRI volumes and ctDNA levels, rather than ICD induction or side effects as previously reported. Changes during treatment might predict clinical disease course.

## Introduction

For locally advanced rectal cancer neoadjuvant long-term chemoradiotherapy (NCRT) with 5-fluorouracil (5-FU) followed by surgery is the standard of care.^1,2^ Besides, emerging strategies and intensified regimes like total neoadjuvant therapy (TNT) and organ preservation are gaining attention.^3–5^ However, the prediction of tumor response remains challenging as some patients achieve pathologic complete response (pCR) after NCRT leading to improved outcomes^6^ whilst others show only moderate tumor regression in spite of identical treatment. Most attempts to identify biomarkers focus on single markers or modalities. In contrast, this project combines imaging with genetic and protein-based blood markers.

High Mobility Group Box 1 protein (HMGB1) is a non-histone protein containing a DNA-binding domain that acts in multiple roles as it can be found in the nucleus as well as in the cytoplasm.^7,8^ Furthermore, HMGB1 can be found in the extracellular space due to active secretion (e.g. by lysosomes in case of cellular stress) or passive release (e.g. necrosis, apoptosis) and has been reported to imply both, pro-inflammatory and anti-inflammatory signaling.^8,9^ In particular, the significant immunostimulatory role of HMGB1 as a damage-associated molecular pattern (DAMP) released during immunogenic cell death (ICD) triggered by anticancer treatment has been described.^10^ Concordantly, HMGB1 has been identified as a surrogate marker for ICD in vitro.^10^

The role of HMGB1 in radiotherapy inheres ambivalence as well.^11^ Extracellular HMGB1 supports complex signalling: on the one hand HMGB1 promotes immunostimulation as one of the major described DAMP.^12^ On the other hand, extracellular HMGB1 promotes immune evasion, chronic inflammation, neo-angiogenesis and tumor invasion mediating radioresistance.^11^

The concentration of serum HMGB1 as a prognostic factor in diverse human cancers has been described before with different implications.^13–15^ Thus, the aim of this study was to closely monitor HMGB1 in the plasma of patients with locally advanced rectal cancer receiving NCRT to analyze dynamics during treatment. Using an integrative approach, magnetic resonance imaging (MRI) volumes (in dedicated MRI scans) and circulating cell-free tumor DNA (ctDNA) levels were analyzed alongside HMGB1. Liquid biopsies detecting ctD-NA have been investigated extensively during the last decade and are considered a valuable biomarker for tumor burden and disease monitoring.^16–18^ ctDNA dynamics during chemoradiation as well as sequencing results of the same cohort were published previously and we observed a correlation of ctDNA with early MRI response.^19,20^

## Patients and methods

### Study design

After approval of the local ethics committee of Tübingen, Germany (734/2015BO2; following the Helsinki Declaration), 19 patients with locally advanced rectal cancer were included in this prospective biomarker study. All participants declared their written informed consent. After initial diagnosis by biopsy, CT-staging to rule out distant metastases and MRI of the pelvis, neoadjuvant treatment comprised long-term radiotherapy (50.4Gy in 28 fractions) and two cycles of concurrent chemotherapy (5-Fluorouracil in week 1 and week 5). Six patients were treated with additional regional hyperthermia. At the end of week 2 and week 5 patients received dedicated MRI scans of the pelvis as part of the research protocol and tumor volumes were delineated using a T2 weighted imaging sequence for radiographic early response evaluations. Pathologic response of the resection specimen after NCRT was described by Dworak regression grade.

For standardization and to rule out potential confounders, blood samples (EDTA tubes, Sarstedt, Nümbrecht, Germany) were preferably collected on Mondays before radiotherapy in a weekly manner. The baseline sample was drawn before treatment start on day 1.

### HMGB1 and genetic testing

Plasma was separated by dual centrifugation and stored at -80°Celsius to prospectively analyze HMGB1 and circulating cell-free tumor DNA (ctD-NA) as previously described.^19,21^

HMGB1 was measured by Enzyme-linked Immunosorbent Assays (ELISAs) as indicated by the manufacturer’s instructions (IBL International GmbH, Hamburg, Germany, Reference Number ST51011). Standard curves were acquired and plasma samples were analyzed in technical duplicates. Means were calculated to enhance precision of data as described before.^21^

Primary tumors were sequenced by a dedicated cancer panel including 708 cancer genes. ctDNA was analyzed by a tumor-informed approach were using individualized capture panels aiming on ultra-deep sequencing. Sequencing data was error-corrected during deduplication (using unique molecular identifiers – UMIs) and only reads with at least three duplicates were kept. The mean allele frequency was calculated based on the patient-specific variants. Dedicated methods of genetic sequencing and bioinformatic evaluation have already been reported.^19,20^

### Statistics

For statistical analysis we used IBM SPSS version 28. HMGB1 levels were evaluated as absolute values and as relative dynamics (normalized to the concentration of the baseline sample of day 1). Correlations were calculated by the Mann-Whitney U-Test, the Kruskal-Wallis-Test and the two-sided Pearson correlation. For median definitions, patients were considered if both metrics were available. When correlating the initial HMGB1 and MRI volume, all 19 patients could be evaluated. Patient 107 who represented the median of HMGB1 (9.4 ng/ml) was counted for the group ≤ median. Pathologic response was recorded using the Dworak regression grade: Dworak 1 or 2 was defined as poor response and Dworak grade 3 or 4 was recorded as good response. P-values ≤ 0.05 were considered statistically significant. P-values ≤ 0.1 were reported as trends due to the limited cohort size.

## Results

Three female and 16 male patients could be evaluated in this study. Mean age was 63 years at diagnosis (range 37–79 years). Median follow-up was 54 months. Therapeutic side effects at the time of blood sampling were moderate (CTC 1–2°) with only one CTC grade 3 toxicity (diarrhoea requiring parenteral support) as shown in Table 1. We did not find a correlation between HMGB1 courses and these (moderate) therapeutic side effects (Table 1).

**TABLE 1.** Side effects during chemoradiation and HMGB1

Patient	Toxicity (CTC)	Infection / complication	HMGB1 during treatment
101	1° (skin)	upper extremity venous thrombosis	undulation / ascent***
102	1° (skin)	urinary tract infection	undulation / ascent***
103	2° (skin)		undulation / ascent***
104	2° (skin)		undulation / ascent***
105	3° (proctitis, diarrhea)		undulation / ascent***
106	1° (skin)		undulation / ascent***
107	2° (skin)		undulation / decline****
108	2° (diarrhea)	rosacea / psoriasis exacerbation*	undulation / ascent***
109	0		Decline
110	1° (skin)		undulation / ascent***
111	1° (skin)		undulation / ascent***
112	1° (skin)	pulmonary embolism (after week 4**)	undulation / ascent***
113	0		undulation / ascent***
115	2° (skin)		Ascent
116	1° (skin)		Ascent
117	1° (skin)		decline
118	1° (skin)		undulation / ascent***
119	2° (skin)		ascent
120	2° (skin)		ascent

1 * = during chemotherapy, spontaneous remission;

1 ** = no more study samples were collected after this event;

1 *** = undulation with ascent towards the end of treatment;

1 **** = undulation but decline at the end of therapy

**FIGURE 1.**
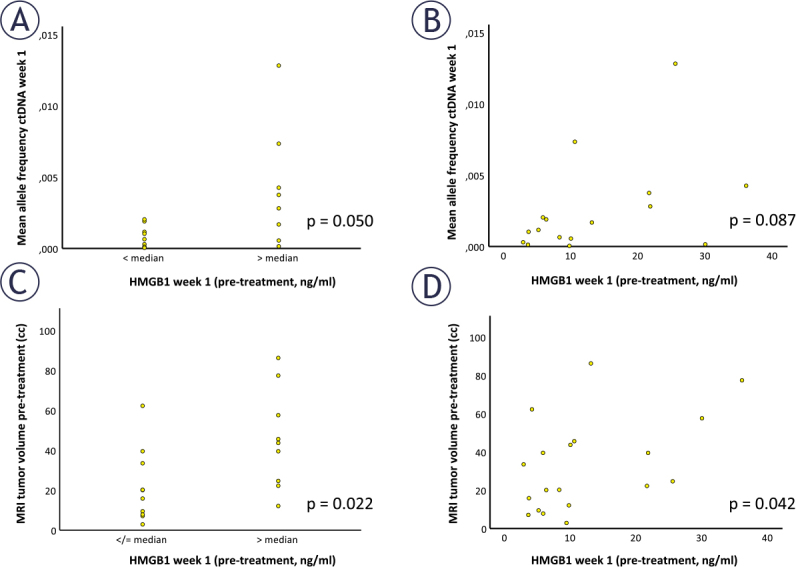
Initial High Mobility Group Box 1 protein (HMGB1) levels of the pre-therapeutic sample on day 1 of treatment were correlated with the corresponding mean allele frequency (AF) of cell-free tumor DNA (ctDNA) grouped according to the median of HMGB1 **(A)** and absolute values **(B)**. In Figure 1C and 1D HMGB1 levels were correlated with the initial tumor volumes in magnetic resonance imaging (MRI), respectively.

In total, we analyzed 101 plasma samples to measure HMGB1 during NCRT. Initial HMGB1 measures ranged from 2.9 ng/ml to 36.1 ng/ml (median 9.4 ng/ml). Using median dichotomization, we found high levels of HMGB1 to correlate significantly with elevated mean allele frequency of ctDNA (p = 0.05, Figure 1A). A trend toward a significant positive correlation could be observed between individual values of HMGB1 concentrations and corresponding mean allele frequency of ctDNA (Figure 1B). Initial tumor volumes were also significantly larger in patients with HMGB1 concentrations above the median (Figure 1C), and MRI volumes correlated significantly with individual HMGB1 concentrations (Figure 1D).

**FIGURE 2.**
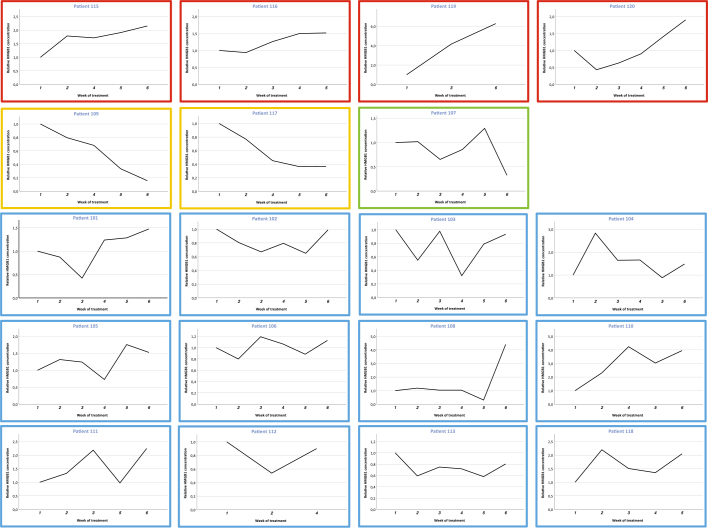
Individual relative dynamics of High Mobility Group Box 1 (HMGB1; normalized to the initial concentration) during the course of neoadjuvant chemoradiotherapy. Four patients showed mainly ascending values of HMGB1 (indicated in red boxes), two patients had HMGB1 decline (yellow frame), one patient showed undulating levels but decline at the end of therapy (green) and several patients had undulating HMGB1 concentrations in the plasma with ascent towards the end of treatment (blue).

Relative dynamics of HMGB1 during treatment (normalized to the individual pre-therapeutic value) showed various patterns (Figure 2). We identified four groups (descriptive): predominant elevation / decline of HMGB1 values and undulating values with ascent / descent towards the end of therapy. Most patients showed undulating levels of HMGB1 with ascent towards the end of treatment (week 5 / 6). However, one patient presented with an undulating course of HMGB1 and decline at the end of NCRT (pat. 107). This patient had the smallest initial tumor volume and achieved pCR. In four patients, mainly ascending curves were observed (pat. 115, 116, 119 and 120). Of these, all had poor pathologic response (Dworak regression grade 1 or 2) and two patients suffered from metastatic disease during follow-up (pat. 116 and 119). In contrast, two patients showed descending HMGB1 levels during treatment (pat. 109 and 117). These patients had initially high absolute HMGB1 measures (pat. 109: highest HMGB1 in the cohort (i.e. 36.1 ng/ml) and pat. 117 third highest initial HMGB1 level (i.e. 25.5 ng/ml)), and the highest (pat. 117) and third highest (pat. 109) baseline AF. In addition, patient 109 presented with the biggest primary tumor volume in our cohort.

**FIGURE 3.**
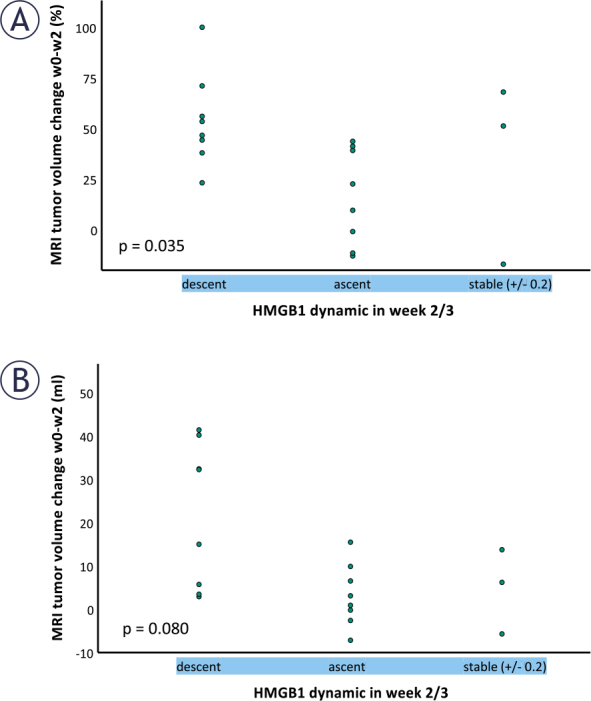
The dynamics of High Mobility Group Box 1 (HMGB1) measures were compared to the corresponding magnetic resonance imaging (MRI) at the end of week 2 of neoadjuvant treatment. We evaluated dynamics at the beginning of week 3 or - if not available, considered week 2. Levels normalized to the pre-treatment concentrations were considered as “stable” if they were in the range of + / - 0.2. Patients with decline of HMGB1 values showed enhanced relative MRI tumor regression (%) **(A)** and a trend regarding absolute tumour volume decrease **(B)**.

For early response evaluation, we analysed volumetric MRI response at the end of week 2 and the respective HMGB1 dynamics relative to baseline in week 3. If this value was not available, we used the value of week 2. We grouped patients according to ascent, descent or stable values (> 1.2; < 0.8; 0.8–1.2, respectively) as shown in Figure 3A. Patients with decreasing HMGB1 levels in week 2/3 showed a greater percentage reduction of MRI tumour volumes compared to those with increasing HMGB1 levels (p = 0.030; p-value comparing all three groups = 0.035). Considering absolute volume decrease in MRI (ml) similar dynamics with a trend to significance were found (p = 0.080; Figure 3B).

If patients showed lower absolute HMGB1 levels in the last collected plasma sample a trend to better Dworak response (Dworak 3 or 4) was found (median HMGB1 level for patients with good response: 8.4 ng/ml; median HMGB1 level for patients with poor response: 11.8 ng/ml; p = 0.091). However, we did not find significant correlations of HMGB1 dynamics and long-term outcome in this small cohort (data not shown).

## Discussion

This is the first report correlating HMGB1, as a possible marker for immunogenic cell death, with consecutive MRIs during treatment and ctDNA levels in the same patient cohort. Thus, biological markers on a protein level could be evaluated in the context of genetic data (ctDNA), imaging data and clinical outcome (pathologic response and recurrences) of NCRT for rectal cancer.

Especially in the preclinical setting, HMGB1 release to the extracellular space has been identified as one of the consensus markers for immunogenic cell death.^10^ Extracellular HMGB1 triggers the activation of the innate immune system. In 2012, the elevation of HMGB1 in the serum of patients with esophageal cancer undergoing preoperative chemoradiotherapy was interpreted as immunogenic cell death induction.^22^ However, the regulation of HMGB1 levels and changes during and after oncologic treatment seems to be much more complex.

The half-life time of HMGB1 seems to be rather short, even though the rate of degradation is dependent on the extracellular environment and different redox states. A range of 17 minutes (reduced state: all-thiol HMGB1, in serum), three hours (all-thiol HMGB1 in extracellular fluids of prostate cancer) and 10–11 hours (disulfide HMGB1 in serum) of half-life time was reported.^23^ Thus, we consider HMGB1 as a suitable biomarker to reflect recent tumour changes during long-term NCRT. The consideration of relatively short half-life of HGMB1 might support the correlation of enhanced tumour regression in MR imaging (week 2) and relative decrease of HMGB1. Of note, the observed undulating trends of HMGB1 (Figure 2) may be partly explained by irregular peaks of tumuor cell death, apoptosis and necrosis during the course of treatment which might cause temporarily elevated HMGB1 levels. As blood samples were collected after the weekend, we intended to avoid detecting a potential rise in HMGB1 following radiotherapy sessions. The goal was rather to track intermediate changes due to tumour size decrease during 5-week-chemoradiotherapy.

During definitive chemoradiotherapy of head and neck cancer, HMGB1 levels were associated with treatment-induced toxicity and infections, which are discussed as possible further confounders.^21^ However, high grade toxicity associated with cell death in normal tissue (≥ grade 3) is rare during NCRT for rectal cancer, as confirmed in our cohort. This might explain the different HMGB1 dynamics and different correlations in the two patient cohorts.

In the pretreatment setting, HMGB1 correlated with the pre-therapeutic MRI tumour volume, as well as ctDNA abundance (AF). Thus, HMGB1 seems to be related to tumour burden before the start of therapy. This might reflect higher rates of spontaneous tumour cell death in larger tumours. Similar findings were reported in head and neck cancer before definitive chemoradiotherapy with a significant correlation of initial HMGB1 levels and gross tumour volume contoured for radiotherapy.^21^ In a cohort of patients with gastric pathologies, HMGB1 was associated with malignancy.^24^

We detected various patterns of HMGB1 dynamics during treatment. In most patients, values were undulating. However, in four patients predominantly ascending values were observed. Notably, these patients all exhibited poor pathological response in the resection specimen, and two of them developed metastatic disease. Similar associations were found in patients with diverse tumor entities undergoing oncolytic virus therapy.^25^ In hepatocellular carcinoma, non-descending HMGB1 levels after sorafenib or hepatic arterial infusion chemotherapy were also correlated with limited prognosis.^26^

Limitations of our study are the pilot character, the descriptive allocation of dynamics in Figure 2 and the limited number of patients. However, weekly sampling and the relevant total number of samples allow first interesting insights into HMGB1 dynamics of the individual patients during long-course chemoradiation for rectal cancer.

In conclusion, in the setting of NCRT for rectal cancer, pre-therapeutic HMGB1 appears to be primarily correlated with tumor volume as well as respective ctDNA levels. Changes during treatment might correlate with clinical course and oncologic outcome.
